# Dysbiosis of the gut and lung microbiome has a role in asthma

**DOI:** 10.1007/s00281-019-00775-y

**Published:** 2020-02-18

**Authors:** Karin Hufnagl, Isabella Pali-Schöll, Franziska Roth-Walter, Erika Jensen-Jarolim

**Affiliations:** 1 The Interuniversity Messerli Research Institute, Medical University Vienna and University of Veterinary Medicine Vienna, Vienna, Austria; 2 Center for Pathophysiology, Infectiology and Immunology, Institute of Pathophysiology and Allergy Research, Medical University Vienna, Währinger G. 18-20, 1090 Vienna, Austria

**Keywords:** Asthma, Allergy, Microbiome, Th2 inflammation, Antibiotics, Probiotics

## Abstract

Worldwide 300 million children and adults are affected by asthma. The development of asthma is influenced by environmental and other exogenous factors synergizing with genetic predisposition, and shaping the lung microbiome especially during birth and in very early life. The healthy lung microbial composition is characterized by a prevalence of bacteria belonging to the phyla *Bacteroidetes*, *Actinobacteria*, and *Firmicutes*. However, viral respiratory infections are associated with an abundance of *Proteobacteria* with genera *Haemophilus* and *Moraxella* in young children and adult asthmatics. This dysbiosis supports the activation of inflammatory pathways and contributes to bronchoconstriction and bronchial hyperresponsiveness. Exogenous factors can affect the natural lung microbiota composition positively (farming environment) or negatively (allergens, air pollutants). It is evident that also gut microbiota dysbiosis has a high influence on asthma pathogenesis. Antibiotics, antiulcer medications, and other drugs severely impair gut as well as lung microbiota. Resulting dysbiosis and reduced microbial diversity dysregulate the bidirectional crosstalk across the gut-lung axis, resulting in hypersensitivity and hyperreactivity to respiratory and food allergens. Efforts are undertaken to reconstitute the microbiota and immune balance by probiotics and engineered bacteria, but results from human studies do not yet support their efficacy in asthma prevention or treatment. Overall, dysbiosis of gut and lung seem to be critical causes of the increased emergence of asthma.

## Introduction

Asthma nowadays affects an estimated number of more than 300 million people worldwide and incidences are still increasing. The symptoms include mainly narrowing and inflammation of the airways which seem to originate from a synergy between environmental and genetic factors. The pathogenesis of asthma during childhood can be correlated to factors such as delivery by cesarean section, use of antibiotics during the neonatal period, maternal diet low in fiber, milk formula feeding, and the variety of microbes due to environmental exposure [1, 2]. The human body harbors an enormous number of microbes, such as bacteria, viruses, and fungi, that live on its internal and external surfaces. Evidence is accumulating that microbial colonization of mucosal tissues during infancy are important for the development, maintenance, and control of the immune system [3]. It has been recognized that especially a broad bacterial diversity is critical in maintenance of the immune balance. Bacterial species diversity can be measured within samples (alpha-diversity), with different measurements describing the alpha-diversity as do, e.g., Shannon or Simpson index, and between samples (beta-diversity) (Table 1) [4]. Dysbiosis, i.e., microbial imbalance, especially of the gut microbiota, has been associated with the development of several diseases, including allergic diseases and asthma [5]. For the longest time, the lung was believed to be sterile but numerous publications have revealed that it harbors its own microbiota [6, 7]. Consequently, also lung microbial dysbiosis could have a causative role for the development of respiratory diseases such as asthma.

**Table 1:** Definitions of terms used in this work

Term	Definition
Microbiome	Collective genomes of all microorganisms associated with the human body
Microbiota	Microorganisms (bacteria, viruses, fungi, protozoa) populating the inner and outer surfaces of the human body
Dysbiosis	Imbalance of the microbial community and loss of microbial diversity
16S rRNA gene	Component of the 30S subunit of prokaryotic ribosomes used for bacterial classification
Next-generation sequencing (NGS)	High-throughput technologies allowing rapid parallel DNA sequencing
Alpha diversity	A measure of the richness (how many?) and the evenness (how different?) of bacterial species in a sample
Beta diversity	A measure of the similarity of the bacterial composition between samples/individuals
Gut-lung axis	Bidirectional communication pathway between gut and lung
Atopy	Genetic predisposition to develop allergic hypersensitivity reactions
Probiotics	Live microorganisms that provide health benefits

In this review, we will emphasize and discuss the current knowledge on the association of the human lung microbiome with asthma development and severity, with special focus on environmental factors influencing the lung microbiota composition affecting the gut-lung axis in health and disease.

### The human lung—a sterile environment?

The role of the gut microbiome in the pathogenesis of asthma has been extensively studied and reviewed in the last decades [8, 9]. This is facilitated by non-invasive access to samples that contain high bacterial biomass. In contrast, the microbial density in the lung is much lower, as the human lung harbors around 2.2 × 10^3^ bacterial genomes per cm^2^, which is a factor of 10^2^ less than in the gut [10].

**Fig. 1:**
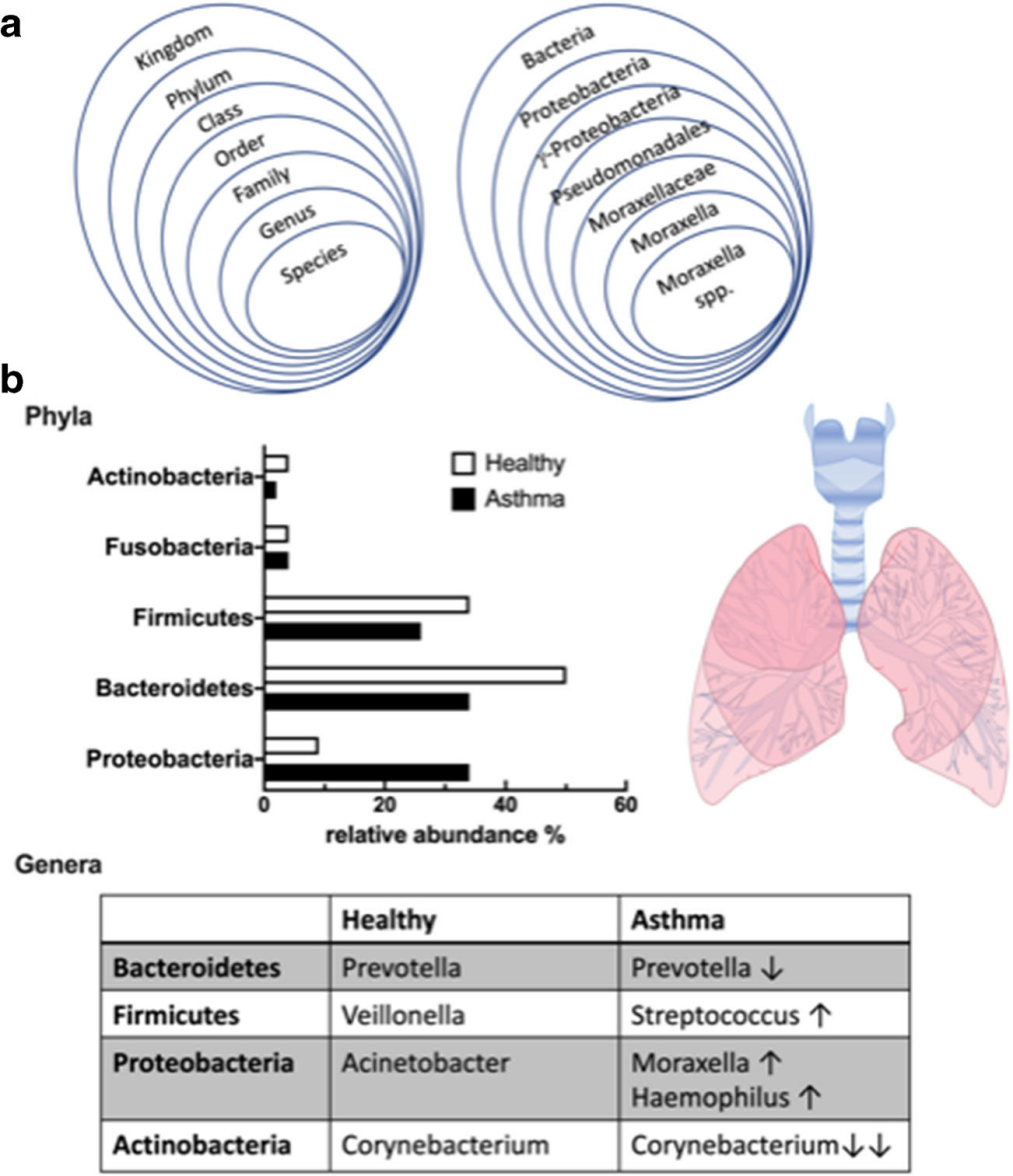
**A** Bacterial taxonomy: classification of the organisms in a rank-based classification (left) and exemplary taxonomical classification of *Moraxella ssp* according to bacterial taxonomy (right). **B** Distribution of common phyla and genera in the airways of healthy and asthmatic subjects: The graph depicts the relative abundance (in %) of the five most common phyla of bacteria colonizing the human airways and lung in healthy (white bars) and in asthmatic (black bars) subjects. Phyla *Actinobacteria, Firmicutes* and *Bacteroidetes* are less abundant in airways of asthmatics, while *Proteobacteria* are enriched. The table includes bacterial genera that seem to have a growth advantage in asthmatic airways, such as *Moraxella* and *Haemophilus* from *Proteobacteria.* In contrast, some genera are less abundant in asthmatics such as *Prevotella* and *Corynebacterium*, leading to a dysbiosis of the airway microbiome

The Human Microbiome Project was initiated to study function and diversity of the healthy human microbiome [11]. Assuming that the lung is sterile, lung tissue samples from healthy participants were not included in this project. The use of next-generation sequencing (NGS) (Table 1), which relies on sequencing and analysis of bacterial 16S ribosomal RNA [12], instead of traditional culture isolation techniques, modernized this field of microbiology and led to frequent detection of bacterial communities in healthy lower airways [13]. The first application of NGS on lower airway samples compared the composition of lung microbiota from healthy and asthmatic adults and children [14]. This study showed that bacteria from the phylum *Bacteroidetes*, and in particular the species *Prevotella*, were more common in healthy than in asthmatic subjects. These microorganisms belong to the group of gram-negative anaerobic bacteria that are not easily cultivated. Further studies documented that also the phyla *Firmicutes* (species *Streptococcus*), *Proteobacteria* (species *Acinetobacter*), and *Actinobacteria* (species *Corynebacterium*) are most abundant in the lungs of healthy individuals [15–17] (Fig. 1). These bacterial phyla are still present and abundant in asthmatic individuals, but there seems to be a shift towards certain genera within these phyla that could generate a dysbiosis (Fig. 1).

The colonization of the human lung with microorganisms is tightly connected with its anatomy and physiological function. Respiratory microorganisms entering the oral cavity arrive at the lung suspended in air or on micro particles. The upper airways are layered with the cylindrical respiratory epithelium covered by a mucous film. The constant fluctuation of mucous fluid and air flow determine the balance between microbial immigration and elimination. Elimination of microorganisms is supported by mucociliary micro-movements and cough, all influenced by host immune status [18]. With transition from the upper to the lower respiratory tract, the gradients for pressure and temperature change and may favor bacterial growth of some bacterial communities by creating anaerobic zones. The smallest lung units, the alveoli at the end of the bronchial tree, are composed of type I pneumocytes, a thin squamous epithelial cell layer, and type II pneumocytes producing a pulmonary surfactant [19]. Surfactant consists of phospholipids (90%) and proteins such as surfactant proteins A to D, with a major innate role in bacterial and viral clearance [20].

It has been proposed that the main source for lower airway colonization is the resident microbiome of the upper airways. It seems also plausible that bacteria may reach the lower airways through microaspiration of oropharyngeal secretions, and to a lesser extent through direct inhalation [16]. Not only the oropharynx but also the nasopharynx is described to be a source of aspirated microbes, especially in children with increased production of nasal secretions [17, 21]. These findings altogether support an ecological model with the composition of the respiratory microbiome being determined by several factors: (1) microbial immigration by microaspiration from the upper airways together with microbial elimination through coughing; (2) mucociliary clearance; (3) both adaptive and innate host defense; and (4) bacterial growth conditions such as pH, oxygen tension, concentration of nutrients, and presence of inflammatory cells. In healthy individuals, a low density and continual renewal of the lung microbiome with low bacterial replication rate has been observed.

Conditions favoring enhanced replication and persistence of certain bacterial species could induce an imbalance or dysbiosis of the lung microbiome, possibly resulting in asthma development.

### The airway microbiome in children: influence on asthma development

The colonization of the upper airways starts very early on, as tracheal aspirates of neonates taken only several hours after birth showed that the predominant phyla were *Firmicutes* and *Proteobacteria*, in addition to *Actinobacteria* and *Bacteroidetes* [22]. It is of interest that the development of the resident respiratory microbiome depends very much on the exposure in the first few hours including delivery mode, and on the environment during the following 4 to 5 months [23–25]. A strong association was also observed between childhood asthma and respiratory infections, mainly induced by human rhinovirus and respiratory syncytial virus [26, 27]. This is often accompanied by altered microbial spectra as shown in a mouse model of viral lung infection, resulting in an increase of phylum *Bacteroidetes* with a concomitant decrease in phylum *Firmicutes* [28].

The microbiome of the upper respiratory tract is accessible even in infants and has been investigated in many studies in the context of asthma development or already established asthma phenotypes in children, in particular, as the upper airway microbiota seems to be the main contributor to the lower airway composition [29]. In this respect, nasal secretion samples from asthmatic children from 6 to 17 years of age showed a distinct microbiota composition dominated by genus *Moraxella* which was associated with increased exacerbation risk and activation of eosinophils [30]. In the same study*,* in vitro testing with *Moraxella catarrhalis* revealed that this bacterium is able to induce epithelial damage and inflammatory cytokine expression (IL-33, IL-8) [30] (Table 2).

**Table 2:** Bacterial genera associated with microbial dysbiosis and asthma

Subjects	Microbiota linked to asthma	Reference
**Airway microbiome**		
Asthmatic children, nasal microbiome	Increased abundance of *Moraxella catarrhalis*; induction of epithelial damage and inflammatory cytokine expression (IL-8, IL-33)	[30]
Children with respiratory disease, nasopharyngeal microbiome	Increased abundance of *Moraxella* and *Haemophilus*; associated with increased risk of wheeze at the age of 5	[31]
Preschool children with severe wheeze, lower airway microbiome	Genus *Moraxella* dysbiosis was associated with airway neutrophilia	[32]
Asthmatic and healthy adults, bronchial brushings	Asthmatic status associated with increased abundance of *Proteobacteria,* especially *Haemophilus*	[14]
Adults with severe asthma	Increased abundance of *Proteobacteria*; associated with Th17-related genes	[33]
Asthmatic and healthy adults, BAL samples	Increased abundance of *Haemophilus parainfluenza*; associated with activation of TLR4, proinflammatory IL-8, inhibition of corticosteroid-related pathway	[34]
Asthmatic and healthy adults, bronchial epithelial brushings	Increased abundance of *Proteobacteria* with *Haemophilus* and *Neisseria* in asthmatics; general lower bacterial diversity associated with high Th2-related lung inflammation	[35]
**Gut microbiome**		
Asthmatic and healthy children, gut microbiome	*Clostridium difficile* colonization at 1 month associated with asthma at the age of 6 years	[36]
Infants at risk for asthma, gut microbiome	Decreased relative abundance of genera *Lachnospira, Veillonella, Faecalibacterium, Rothia* in infants at risk	[23]
Preschool age asthmatic and healthy children, gut microbiome	Decreased relative abundance of genus *Lachnospira,* increased relative abundance of genus *Clostridium neonatale* in asthmatic children	[37]
Preschool age asthmatic and healthy children, gut microbiome	Lower abundance of genera *Faecalibacteria, Roseburia,* increased abundance of genus *Clostridium* in asthmatic children	[38]
Infants at high risk for asthma, gut microbiome	*Lactobacillus rhamnosus*–associated fecal products promote expansion of T-regulatory cells and IL-10 production ex vivo, promoting tolerance	[39]

The relationship between the nasopharyngeal microbiome, the occurrence of acute respiratory infections and early allergic sensitization has recently been analyzed in 244 infants through their first 5 years of life [31]. The dominant bacterial genera in the first 2 years of life of these children were composed of the genera *Moraxella*, *Streptococcus*, *Corynebacterium*, *Alloiococcus*, *Haemophilus*, and *Staphylococcus*, all belonging to one of the phyla *Firmicutes*, *Proteobacteria*, or *Actinobacteria.* Lower respiratory illness at this age was positively associated with *Moraxella*, *Streptococcus*, and *Haemophilus*, while a negative correlation was seen with *Corynebacterium*, *Alloiococcus*, and *Staphylococcus* (Fig. 1). Especially, respiratory illness–associated *Moraxella* seems able to destabilize the bacterial respiratory balance by producing biofilms that enhance co-survival of pathogens such as *Streptococcus pneumoniae* and *Haemophilus influenzae* [40]. Additionally, it was shown that in children with early allergic sensitization, the colonization of the upper airways with *Moraxella*, *Streptococcus*, and *Haemophilus* increased the risk of chronic wheeze at 5 years of age. Enhanced allergen-specific IgE levels of these early sensitized children could already be detected at 6 months of age [31] (Table 2).

The aforementioned study corroborates studies that were undertaken more than 10 years earlier by Bisgaard et al. [41]. Hypopharyngeal aspirates from children of the Copenhagen Prospective Study on Asthma in Childhood cohort were cultured and analyzed for bacterial diversity, with positive results for *Moraxella catarrhalis*, *Haemophilus influenzae*, and *Streptococcus pneumoniae*. In children born to asthmatic mothers, the presence of the bacteria was associated with increased risk of wheezing and asthma at 5 years of age [41]. Thus, a dysbiotic nasopharyngeal microbiome seems to be associated with the frequency of recurrent viral infections and asthma development very early in life. Especially, an early predominant *Moraxella* spp. colonization of the upper respiratory tract of children seemed to promote and even precede respiratory tract infections as demonstrated in two independent prospective birth cohort studies [42, 43] (Fig. 1). In this respect, the role of environmental factors in the colonization process of the respiratory tract should not be underestimated. The comparison of the upper respiratory tract microbiome of children living on a farm and nonfarm children revealed an enhanced *Moraxella* species abundance in both groups, but the association of asthma with *Moraxella* colonization was restricted to nonfarm children that are exposed to a much lower diversity of microorganisms [44].

Accessing the lower airways directly is a difficult approach, even more in children [45]. A recent study on preschool children with severe wheeze undergoing fiberoptic bronchoscopy and bronchoalveolar lavage revealed two distinct groups of lower airway microbiota composition [32]: one was a cluster with *Moraxella* species dysbiosis associated with airway neutrophilia; the second represented a mixed cluster with higher diversity of *Streptococcus*, *Prevotella*, and *Neisseria* species, similar to healthy lung microbiota cluster but still associated with a macrophage- and lymphocyte- predominated inflammatory profile [32] (Table 2).

In summary, these findings suggest a window of opportunity to manipulate the upper airway microbiota and, hence, the immune system for preventing the development of asthma in children, although the underlying mechanisms are not fully understood. But there may even be a protective role of the lung microbiome against asthma, consisting of early colonization with a varied, non-pathogenic bacterial community that is not prone to induce Th2 inflammatory responses but rather promotes tolerance.

### The lung microbiome associated with established asthma and atopy in adults

The respiratory microbiome in adults with established asthma has a lower bacterial diversity compared to healthy subjects and an increased richness, both correlating to asthma severity [46]. Several groups have reported an increased abundance of the phylum *Proteobacteria*, and in particular the genus *Haemophilus* in asthmatics [14, 47]. Additionally, Huang and colleagues found that higher abundance of *Proteobacteria* was related to lower asthma control and asthma exacerbations which was accompanied by the induction of Th17-related genes [33]. Especially, enrichment of *Haemophilus* and *Moraxella*, both belonging to the class of *Gammaproteobacteria*, were associated with severe airway obstruction and airway neutrophilia [48, 49]. Most of the mechanistic studies on the influence of airway microbiome on asthma development were conducted in murine models [5, 10]. One of the few mechanistic approaches in human adult asthma related the finding of increased *Haemophilus parainfluenzae* with in vitro studies [34] (Table 2). The authors could demonstrate that *Haemophilus parainfluenzae* was able to activate Toll-like receptor (TLR) 4, which subsequently led to transcription of pro-inflammatory factors such as IL-8 and at the same time inhibited corticosteroid-related pathways. The induction of corticosteroid resistance is an important factor as corticosteroid treatment is one of the mainstay treatments in asthma and inflammation.

Durack and colleagues investigated whether an atopic status could be correlated with alterations in the airway microbiome by comparing patients with mild atopic asthma, atopic patients without asthma, and healthy non-atopic controls [50]. In general, patients with high Th2—associated lung inflammation displayed lower bacterial diversity in the airways. *Proteobacteria*, with genera *Haemophilus* and *Neisseria*, were again enriched in asthmatics, but also the genera F*usobacterium* and *Porphyromonas* were detected, which have earlier been correlated with bronchial hyper-reactivity [35]. Bacteria from the family *Lactobacillaceae*, which seem to be important for regulatory T cell development [39], were reduced. Bacterial dysbiosis associated with atopy, but not with asthma, included members of *Pasteurellaceae*, with *species Aggregatibacter*, but also *Prevotella* ssp. from *Bacteroidetes* and *Corynebacterium* from *Actinobacteria*. These data indicate a bacterial taxonomic overlap with subsets that seem to be characteristic for atopy alone, leading to the speculation that in allergen-sensitized individuals the mucosal lung environment allows for colonization of different microbiota compared to non-sensitized subjects.

Thus, the most common changes in lung microbiota in asthmatics relate to a dysbiosis which favors growth conditions of *Proteobacteria*, in particular of the genera *Moraxella* and *Haemophilus* (Fig. 1b), which may lead to activation of inflammatory Th2 pathways and contribute to bronchoconstriction and bronchial hyper-responsiveness.

### The role of the gut-lung axis in airway microbiome and asthma

Several studies have linked a dysbiosis of the gut microbiome early in life with an enhanced risk for asthma development later in life. Children that developed asthma at school age displayed a lower gut microbiome diversity up to 1 month of age, compared to non-asthmatic children [51]. Colonization by species *Clostridium difficile* (phylum *Firmicutes*) at 1 month of age was associated with wheeze and asthma at the age of 6 to 7 years [36]. Another study analyzed the gut microbiome of infants at risk for asthma in the first 100 days of life and discovered that the relative abundance of the genera *Lachnospira*, *Veillonella*, *Faecalibacterium* (phylum *Firmicutes*), and *Rothia* (phylum *Actinobacteria*) was significantly decreased in these children [23]. This bacterial dysbiosis of the gut microbiome was confirmed in another study by the same group of authors, showing that opposing shifts in the relative abundance of *Lachnospira* and *Clostridium neonatale* in the first 3 months of life was associated with asthma development at preschool age [37] (Table 2). In a recent metabolomics-based approach, stool samples from children aged 4 to 7 years with asthma were compared to healthy children, with a focus on gut metabolites such as amino acids or butyrate [38]. Taxonomic classification showed that among gut bacteria, the phyla *Firmicutes* (67.8%), *Actinobacteria* (20.7%), and *Bacteriodetes* (8.4%) accounted for 97% of all analyzed sequences. Children with asthma presented a significant lower abundance of genera *Faecalibacterium* and *Roseburia* (phylum *Firmicutes*), while genera *Enterococcus* and *Clostridium* (phylum *Firmicutes*) were enhanced compared to healthy controls. Bacterial dysbiosis within the phylum *Firmicutes*, which were of significantly lower abundance in children with asthma, could therefore be related to an increased asthma risk. Thus, considering the risk for asthma, a relation to lung *and* gut microbiome can be found. Important factors for the development of the microbiome in both niches encompass an early life window for microbiome establishment, diversity, and richness of bacteria, and effects of the bacteria on the immune system.

There is accumulating evidence that a cross-talk exists between gut and lung, the so-called gut-lung axis, and its importance for maintenance of immune homeostasis has been highlighted [52]. The mechanisms by which the gut microbiota influence microbiota of the lung and vice versa are not fully understood but it seems that intestinal and respiratory diseases display overlapping pathological changes and a shift from gut inflammation to lung inflammation could occur [53]. Thus, disturbances in this bidirectional exchange are linked to increased emergence of airway diseases such as asthma [54]. In accordance, it is evident that patients with chronic gastrointestinal diseases have a higher prevalence of pulmonary diseases [55].

**Fig. 2:**
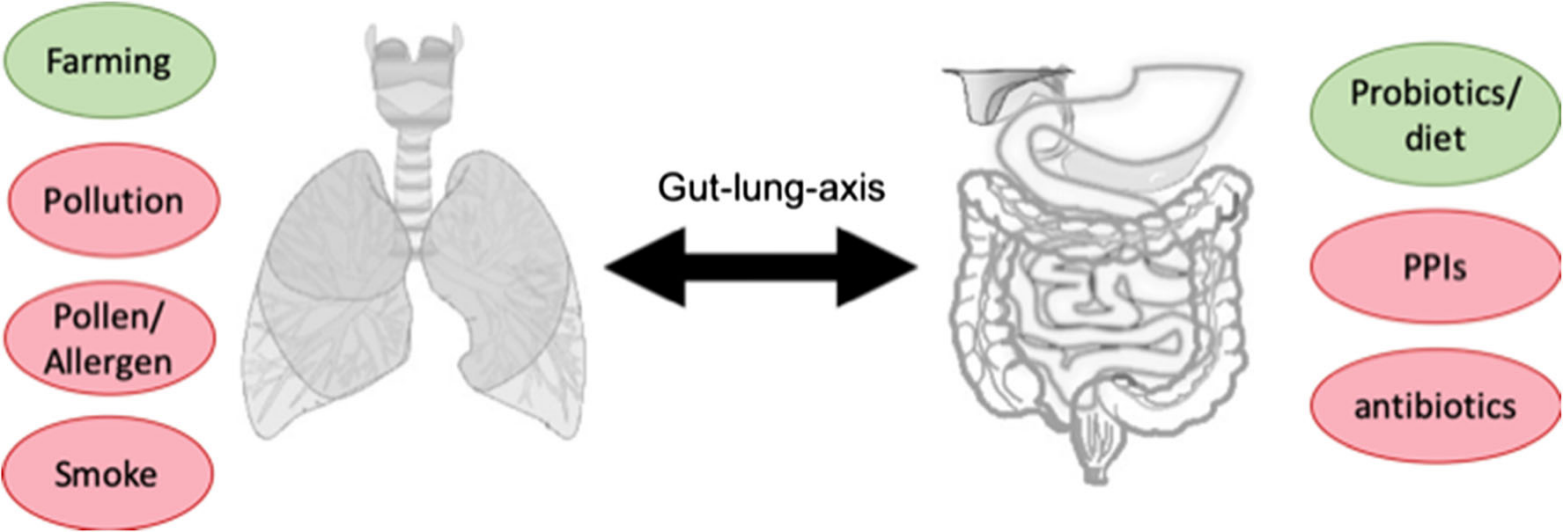
Environmental factors associated with asthma and their influence on the gut-lung axis. Environmental factors can have a positive/protective effect (green circles) or a negative/enhancing effect (red circles) on asthma development. For some of these factors (e.g., antibiotics, pollution), it was demonstrated that they are able to interfere with the gut and/or lung microbiome, leading to dysbiosis and disturbances in the bidirectional exchange via the gut-lung axis, thereby enhancing asthma prevalence. Protective factors such as farming environment or intake of probiotics account for lower asthma incidences, but the direct impact on the gut or lung microbiome still needs to be analyzed in more detail

Environmental factors could have a major impact on asthma, but also preventive or therapeutic measures such as drugs (e.g., antibiotics), probiotics, or bacterial lysates could interfere via the gut-lung axis with the airway microbiome and asthmatic disease (Fig. 2) and will be discussed in the following chapters.

### Environmental factors

Environmental factors like pollen, allergens, and pollutants can cause bacterial dysbiosis and thereby promote asthma. On the other hand, also protective factors may derive from environmental exposure, where not only beneficial bacteria or bacterial components but also specific proteins like lipocalins could be protective against the development of asthma and atopic diseases.

#### I) Influence of farming environment on asthma

Among the many exogenous influences on the respiratory system, the best-described environmental factor with a protective role against asthma and allergy development is the farm effect. Growing up on a farm and consuming raw milk has been shown to reduce the incidence of asthma and allergic diseases by around twofold [56–59]. Especially, cattle farms have shown this protective effect, in contrast to sheep, hares, or rabbits [60]. It became clear that not only the type of animal kept on the farms but also the farming conditions may play a crucial role in this scenario: in the Amish people with more traditional farming conditions, the asthma incidence was four times lower than in the genetically very similar people of the Hutterites, who are technologically much more advanced in terms of dairy farming [61]. To profit from this beneficial effect it is sufficient to live in close proximity to a farm, as growing up within a radius of up to 327 m can protect from asthma and atopy [62, 63]. A difference in the prevalence of asthma and allergic diseases was also found when comparing Amish versus Old Order Mennonites, with the latter being more affected [64]. However, there were only few obvious differences, like smaller farm size and fewer animals per farm for the Amish folk, therefore the divergent prevalence could not reasonably be explained. It has recently been discussed whether asthma in the parent generation might lead to a selective migration, because these asthmatic parents might not like to raise their children on a farm due to high concentrations of endotoxin, organic dust, and microbes. However, a recent paper showed that the asthma-protective effect is not due to selective migration [65].

Also a changed indoor microbiome is may be held responsible for the protective effects, confirmed by the observation that in children who grow up in non-farm homes, asthma risk decreases with an increasing similarity of their home bacterial microbiota composition to that of farm homes [66]. Along these lines, growing up with pets in the first year of life is protective against asthma and allergy development at the age of 6–7 years in children (“mini farm-effect”) [67]. Especially keeping a dog—in contrast to only cats—also reduces the risk in a dose-dependent fashion, probably because dogs and with them their holders are more outdoors and have more, and more diverse contact to microbes. Accordingly, a recent study showed that the dust of homes keeping especially a dog showed a more diverse microbial community than homes without any furred pet [68]. They identified 56 bacterial genera that were significantly more abundant in homes with dogs, which included members of the *Prevotella*, *Porphyromonas*, *Moraxella*, and *Bacteroides* genera, found in mouth and feces of the animals [69]. Subsequently, dog-keeping can also change the microbiome of the owner, who has a skin microbiome more similar to that of the own dog than any other dog [70]. Notably, not only skin but also gut microbiome was influenced by contact with furred pets in infants [71].

Responsible factors for the protective farm effect have been investigated since decades [72]. Among those molecules from cowsheds, which often are derived from bacteria, yeast, or fungi and can activate the innate immune system, endotoxin, a lipopolysaccharide (LPS) , is a compound of special interest from gram-negative bacteria, e.g. *Acinetobacter lwoffii* [73]. Endotoxin was shown to reduce allergen activation of epithelial cells and dendritic cells of airways in a mouse model and also in vitro in human bronchial epithelial cells by inducing the ubiquitin-modifying enzyme A20 [74, 75]. Supporting this observation, in a mouse model where the gene encoding for A20 was selectively knocked out in lung epithelial cells, animals were no longer protected from asthma by LPS pretreatment. In accordance, the median endotoxin levels found in Amish house dust was 6.8 times as high as in Hutterite homes [61]. For the outcome measure “current wheeze” in farm children, exposure to β(1,3)-glucans, and fungal extracellular polysaccharides and endotoxin was also inversely correlated, however, these exposure levels did not explain protection from asthma and/or atopic sensitization [60].

Another molecule from the farm, found to influence the immune system in a positive way, is the N-glycolylneuraminic acid (Neu5Gc), a sialic acid that is produced by non-human mammal cells, but not by bacteria or humans. Children having more anti-Neu5Gc-IgG antibodies were more likely to live on a farm and less likely to suffer from wheeze and asthma [76].

Apart from these bacterial components, specific proteins that stem from animal sources and have innate immune functions seem to play a role. In our working group, we evaluated the effect of carrier-molecules of the secretory lipocalin protein family. By sequestering iron from bacteria, they act bacteriostatic [77] (see also below). Among the lipocalins, the important whey protein beta-lactoglobulin (BLG) from milk has certain specific immune-modulatory effects depending on the loading state: when complexed with iron-quercetin complexes [78], with vitamin A [79], or with zinc (Pali-Schöll et al., ms. in preparation), this molecule exerts an allergy-protective effect, in contrast to being allergenic when empty. Given that almost all mammalian proteins, which can act as allergens, belong to the lipocalin family, we hypothesize that the loading or association state of lipocalins and lipocalin-like molecules may determine the outcome of the immune response in individuals encountering these environmental proteins. Confirming the assumption that lipocalins might contribute to the protective farm-effect, we found the lipocalin BLG also in farm environment in zinc-loaded form (Pali-Schöll et al., ms. in preparation). Importantly, expression of proteins like human lipocalin 2 (LCN2) have been described as microbiota-inducible and MyD88-dependent, stressing their function as innate immune proteins and connecting these important lipocalin molecules to the microbiome [80].

Interestingly, farming environment also exerted a positive effect on children undergoing allergen-specific immunotherapy [81]. In a retrospective analysis of children aged 8 to 16 years, who completed 3 years of subcutaneous immunotherapy due to allergic rhinitis/asthma, those living in a farm area were compared to those having their home in a city. Evaluation of questionnaires showed that living in farm areas was independently associated with significant improvement in total score (average score for symptoms and medication intake) after immunotherapy.

The optimal farm effect for children seems to correlate with a higher number of different animal species encountered already during pregnancy, supporting the expression of receptors of the innate immune system, like TLR2, TLR4, and CD14 [2, 82].

Overall these data suggest that environmental exposure to a varied quantity of not only bacterial components but also proteins could be protective against the development of asthma and atopic diseases.

#### II) Influence of pollen, allergens, and pollutants on airway microbiome and asthma

Specific microbial nutritional requirements as well as environmental factors determine the microbial composition at mucosal sites. Thus, knowledge of the “functional microbiome” where the influence of the microbiome on health and disease is less dependent on one bacterial species in particular, but rather on the presence of microbes capable of fulfilling certain functions, such as the generation of short-chain fatty acids or production of vitamins, is pivotal.

Bacteria, viruses, and fungi from both the upper and lower respiratory tract produce metabolites that interact with the host and alter the development and progression of asthma. It seems that environmental factors are capable to directly interact with these compounds and alter their optimal conditions for growth and their production of microbial compounds and metabolites.

Many aeroallergens belong to specific protein families that can directly interact with microbial constituents, e.g., members of the lipocalin protein family are known to bind to microbial iron-chelators named siderophores [77], thereby directly affecting their virulence and the microbial composition at the mucosal site. Similarly, pollen allergens such as from birch belonging to the pathogenesis-related protein 10 family are components of the innate immunity system in plants and can exert antimicrobial activities at the mucosal linings [83], by capturing iron [84]. As such, allergens may not only trigger asthmatic events by IgE cross-linking, but may alter the microbial composition at mucosal sites per se.

Plants host various bacterial communities on their phyllosphere, which is the microbial habitat defined by the surface of aboveground plant organs. Determination of microbial habitation of 56 tree species using 16S rRNA clone libraries revealed that relative few bacterial phyla define phyllosphere communities: *Actinobacteria*, *Bacteroidetes*, *Firmicutes*, and *Proteobacteria* [85]. Interestingly, the same phyla are dominating in the healthy human lung. In recent years, special attention was paid to bacterial microbiota associated with pollen [86]. Analysis of pollen grains by electron microscopy revealed the presence of single bacterial cells, bacterial clusters, or biofilm-like structures attached to the pollen wall. Four main phyla were characterized from pollen isolates of rye, birch, rape, and autumn crocus with *Proteobacteria* and *Actinobacteria* being more abundant than *Firmicutes* and *Bacteroidetes* [86]. The air-borne pollen of wind-pollinating plants (trees, grasses, weeds) could therefore transport various bacterial species to the human respiratory tract. The allergenic potential of pollen could be explained by simultaneous expression of immunogenic proteins, the allergens, in combination with non-allergenic molecules (e.g., pollen-associated lipid mediators) as well as bacterial adjuvants such as endotoxin derived from gram-negative bacteria [87, 88]. These studies substantiate that allergenicity of pollen could be modified due to microbial or environmental stress factors, in line with the classical concept of pathogenesis—related proteins, which are often not only innate defense molecules of the plant but also allergens. Thus, pollen has its own microbiome and could interfere with allergic sensitization and lung inflammation, resulting in promotion of asthma development.

Air pollution, especially particular matter, is associated with worsening of lung function. The surface of particular matter is rich of metals such as iron, copper, zinc, manganese, and other transition elements, as well as polycyclic aromatic hydrocarbons (PAH), which are able to increase free radical production in the lung, consume antioxidant ingredients, and cause oxidative stress [89]. As such, these particulate substances are able to affect microbial composition and lead to a decrease of the relative abundance of *Bacteroidetes* and *Fusobacteria* alongside with an increase in the relative abundance of *Firmicutes*, *Proteobacteria*, and *Actinobacteria* in the oropharyngeal mucosa [90]. In the respective study, the increase of particulate matter was associated with increased levels of sulfur dioxide (SO_2_), nitrogen dioxide (NO_2_), and ozone (O_3_) which very likely co-contribute to the microbial changes. Similarly, particular matter tobacco smoke results in a greater abundance of *Proteobacteria* in both the feces of smokers and lung brushings of chronic obstructive pulmonary disease (COPD) patients [91, 92]. Tar and irritants contained in tobacco smoke further settles in the moist lining of the airways changing the mucosal and microbial landscape and causing inflammation.

In summary, environmental stress factors such as pollen, allergens, and pollutants can cause bacterial dysbiosis and promote asthma.

### Influence of drugs on microbiota composition

There is a series of drugs that may significantly impair the natural diversity of microbiota. Most logically, antibiotics affect significantly and with immediate and sustained effect the gut microbiota composition, supporting subsequent development of various diseases. In a mouse model, this effect was explained by a change not only of phylotypes and diversity, but also of bacterial metabolites with positive or negative effect on health, even in a gender-dependent manner [93]. Interestingly, antibiotics—like social stress—affect the microbiota composition and lead to microbial translocation into the mesenteric lymph nodes [94].

In a systematic review with the aim to summarize current findings about non-antibiotic gut microbiome changes, according to PRISMA guidelines, however, also non-antibiotic drugs, especially proton pump inhibitors (PPI) and antipsychotic medications were identified as significant causative factors for dysbiosis [95]. Mechanisms affecting microbiota therefore include direct bactericidic or bacteriostatic effects (antibiotics, anti-psychotics like olanzapine), elevation of the gastric pH, and inhibition of peptic digestion like PPIs and other anti-ulcer drugs [96], reduction of blood flow in gut (non-steroidal anti-inflammatory drug (NSAID)), change of transit time (opioids, statins), or modification of inflammation (antipsychotics, statins, opioids, NSAID) [95] (Table 3).

**Table 3:** The effects of drugs on microbiota composition

Category	Drug	Strains affected	Alpha diversity^1^	Beta diversity^2^	Abundance	Organism	Notes	Study reference
Antibiotic	Vancomycin	*Firmicutes* ↓	↓		↓	Mouse, male, female	Colonic, 14 days after treatment	[93]
	Ciprofloxacin-metronidazole	*Firmicutes* ↓	↓		↓	Mouse, male		[93]
		*Bifidobacterium* ↓	↓			Human		[97]
	Vancomycin	*Firmicutes ↑*				Mouse, perinatal treatment	Hypersensitivity pneumonitis unaffected	[98]
	Streptomycin	*Bacteroidetes ↑*				Mouse	Hypersensitivity pneumonitis *↑*	[98]
	Amoxicillin		n.s.			Human	5-day treatment course	[99]
	Azithromycin		↓			Human		[99]
	Cotrimoxazole		n.s.			Human		[99]
	Antibiotics mix	*Lachnospira* ↓	↓			Mouse	Intermittent exposure, 3 cycles. Reduction of pulmonary Tregs	[100]
PPI*		*Firmicutes* ↓/*Erysipelotrichales or Clostridiales*	↓	Altered		Human		[95]
		*Actinomycetales ↑*		Altered		Human		[95]
		*Micrococcaceae ↑*		Altered		Human		[95]
		*Streptococcaceae ↑ /Streptococcus pneumoniae ↑*		Altered	*↑*	Human	Gastric, intestinal, esophageal and oral microbiome; after short-term treatments	[95, [101–106]
		While on PPI: *Lactobacillus* ↓ *Stenotrophomonas* ↓ *Haemophilus ↑*	On PPI: no change after PPI: ↑	On PPI: no change after PPI: ↑	After PPI: changed	Human, children	12 GERD infants treated 18 weeks with PPi. After discontinuation correlation to normal age/nutrition	[107]
		*Lactobacillus*/*L. gasseri subgroup, L. fermentum, the L. reuteri subgroup L. ruminis ↑*					GERD patients, 4, 8 weeks treatment	[105]
		*Faecalibacterium* ↓	n.s.			Human	Long-term use over at least 1 year	[104]
		*Clostridium difficile ↑*						[104]
		*Bacteroidetes* ↓ *Firmicutes ↑* *Species: Holdemania filiformis ↑, Pseudoflavonifractor capillosus* ↓	n.s.	Moderate shift			Long-term use over more than 5 years	[108]
		*Gammaproteobacteria ↑*/*Enterobacter, Escherichia, Klebsiella, Citrobacter*		Altered		Human		[95]
		*Clostridium difficile infection. ↑*			*↑*	Human		[95]
		*Bacterioidales*/*Bifidobacterium ↑*			*↑*	Human		
		*Enterococcaceae ↑*			*↑*	Human		
		Gut commensals *↓,* while oral and upper GI tract commensals *↑*	↓			Human	Paired analysis: 70 monozygotic twin pairs	[101]
		*Smithella*	↓			Mouse	Dominant in responders (vs non-responders) to food allergen	[109]
		*Acetivibrio*	↓			Mouse		[109]
		*Faecalibacterium*	↓			Mouse		[109]
		*Porphyromonadaceae / Tanerella, Barnesiella*	*↑*			Mouse		[109]
Other	Olanzapine		↓		Altered	Mouse	Acts bactericidic	[95]
	metformin	*Bifidobacterium bifidum* ↑	No change		Altered	Human		[95]
		*E.coli ↑*						[97]
	NSAID**		No change		Not altered	Human		[95]
	Opioids	*Staphylococcus ↑* *Enterococcus* ↑	↑		Altered	Human	Due to obstipation	[95]

1 Alpha diversity: microbiota diversity in individual site or sample (one value per sample)

2 Diversity between separate samples

*PPI, proton pump inhibitor

**NSAID, non-steroidal anti-inflammatory drug

#### I) Antibiotics

Generally, it is well accepted that changes of the intestinal microbiota, and of their metabolites, has a systemic impact and determines the severity of atopic dermatitis and the susceptibility to asthma [110], especially when occurring in early life [111], up to 1 year after birth [54, 112]. This was demonstrated in a mouse model where an antibiotic mix reduced T-regulatory cell (Treg) abundance in the lung and supported house dust mite-specific asthma [100]. Thus, the clinical risk association between antibiotics and allergies can be modeled in mice, but not in recombination-activating gene 1-deficient mice, suggesting the antibiotic rather impaired the adaptive immune responses [98].

The use of antibiotics is insufficiently regulated and documented, even though it is secured that antibiotics determine the microbiota composition already during pregnancy and lead to an enhanced risk of atopy, asthma, and more in the next generation [113]. Among antibiotics commonly used by pediatricians, especially azithromycin, but not amoxicillin or cotrimoxazole, led to a severe change of the Simpson’s diversity index [99]. A population-based study revealed that the risk for asthma depends on the antibiotic cycles applied in pregnancy, rising the hazard ratio from 1.15 for one, to 1.51 for three or more courses of treatment [114]. Strikingly, in this study, the use of antibiotics 9 months before to 9 months after delivery posed the same elevated risk for the offspring to develop asthma. The effects on an allergy risk in the offspring were strongest with antibiotics use by the pregnant mother in the third trimester [115]. This is important as the numbers of Cesarean deliveries with high antibiotic use are on the rise, and their substantially altered microbiota have been described [116]. Antibiotics in the first week after birth elevate the risk to develop allergic rhinitis until school age by 1.75 [117].

Much less is known about the influence of antibiotics on airway microbiome composition. Recent investigations in patients with persistent uncontrolled asthma showed that after 1 year of azithromycin treatment, bacterial diversity was decreased compared to asthmatics receiving placebo control [118]. A selective effect, especially on *Haemophilus influenzae* but not on *Moraxella* or *Streptococcus*, was observed, which could be a reason for treatment efficacy. Similarly, azithromycin treatment in COPD patients lowered alpha diversity but did not alter total bacterial burden [119]. The association of lung microbiome alterations due to antibiotics and asthma risk requires further studies. Still, use of antibiotics in infancy is correlated with an at least twofold risk of childhood asthma [120–122]. Thus, international guidelines do not support the use of antibiotics in asthma [123], with some exceptions in asthma exacerbations [124].

#### II) Corticosteroids

Another medication that is recommended for the use in asthma to control airway inflammation are corticosteroids. It seems that patients exhibiting more severe airway obstruction and those who require higher doses of inhaled or oral corticosteroids have higher pathogenic species compared to asthmatic patients with better-controlled disease [125]. Earlier studies reported an increase in *Proteobacteria* in bronchial epithelial brushings from adults with mild to moderate asthma after corticosteroid treatment [14, 33]. These findings were corroborated in other studies, showing that the bronchial microbiota in corticosteroid-responsive asthmatics are changed by corticosteroids, with an enrichment in species *Neisseria*, *Moraxella*, and *Haemophilus*, all associated with asthma [50]. Especially, *Haemophilus* was involved in conferring corticoid resistance [34]. Thus, it seems possible that corticosteroids alter the lung microbiome by promoting the colonization of potentially pathogenic bacterial strains and thereby contributing to corticosteroid unresponsiveness.

#### III) Antiulcer drugs

Antiulcer drugs include various antacids, sucralfate, H2 receptor antagonists (H2RA), and especially proton pump inhibitors (PPIs). All act via the reduction of gastric acid subsequently impairing peptic digestion and pancreatic enzyme release, lowering the thresholds for allergic reactions to food allergens [126]. In a mouse model the risk for anaphylactic reactions after PPI treatments was dependent on the microbiota composition [109].

In humans, the phenomenon of gastric acid suppression is comparable to gastric bypass surgery [127], which also supports allergy to dietary compounds [128]. In a paired analysis between 70 monozygotic twin pairs, PPI intake resulted in a severe dysbiosis [101]. Possibly more important, PPIs and other anti-ulcer drugs produce an immune bias to Th2 via innate and adaptive immune cells [129]. In subjects aged 20, PPIs double the risk for subsequent need for anti-allergy medications, in patients above 60 years multiplying the risk even by a factor of 5 [129].

Studies have shown that already short-term treatments with PPIs, e.g., 4 weeks of once daily administrations of 20-mg esomeprazole [102], substantially alter the microbiota composition, lower its diversity and favor the abundance of *Streptococci*, while reducing *Lactobacillus*, over all affecting the ratio of *Firmicutes* to *Bacteroidetes*. The changes affect from oral, esophageal, gastric to intestinal microbiota and enhance the risk for *Clostridium difficile* infections [103], especially after long-term use [104, 108]. As PPIs are used also in gastroesophageal reflux disease (GERD) in infants, subsequent dysbiosis has been investigated [105, 107] (Table 3).

Antiulcer drugs, which via various mechanisms lead to dysbiosis, synergize with antibiotics in the risk for subsequent allergies, anaphylaxis, allergic rhinitis, and asthma, as demonstrated in a retrospective cohort study with 792,130 children, of whom 7.6% were prescribed an H2RA, 1.7% a PPI, and 16.6% antibiotics during the first 6 months of life [122].

#### III) Reconstituting microbiota after Abx

Studies in germ-free (GF) and antibiotics-treated mice showed that food antigens alone can drive Th2 responses and IgE formation, with a key role of follicular helper cells [130]. Vice versa, the authors showed that successful reestablishment of the healthy microbiota could counter regulate this Th2 immune bias. In accordance, transferal of a “healthy microbiome,” especially *Clostridiales*, but not *Bacteroidales*, provided protection against food allergy in mouse and human studies [131].

The application of probiotics, prebiotics, and synbiotics is especially implemented and accepted after antibiotics therapies, while their beneficial properties only start to be recognized in allergy [132] and atopic dermatitis [133]. Apart from that, also fecal microbiota transplants (FMTs) were tried in settings of long-term antibiotics use. In cystic fibrosis, also with a long-term use of diverse antibiotics, such as azathioprin, and a high risk for dysbiosis and *Clostridium difficile* infections, FMT has been tried recently as a complementary approach [134]. In pouchitis, however, FMT showed low efficacy, likely due to low donor engraftment [135].

Generally, the achievement of persistent colonization is a major hurdle in reconstituting gut microbiota, but could recently be solved by specific probiotic strains. Cesarean section babies are usually treated by antibiotics and are at risk for impairment of the development of a healthy gut microbiome [116]. When a probiotic mixture was fed to antibiotic treated babies, an increase of *Bifidobacteria* and reduction in *Proteobacteria* and *Clostridia* was only observed in those babies fed with mother’s milk, resembling a natural prebiotic [136]. These studies give hope that recolonization can be achieved in the near future.

Taken together, various drugs impair the healthy composition of microbiota, rendering a risk for immune deviation towards Th2 and for diseases like allergies and asthma. Besides other drugs, antibiotics and anti-ulcer drugs, especially PPIs, reduce the Shannon’s diversity index. While antibiotics act directly anti-bacterial, PPIs knockout the physiological gastric bactericidic function. Pregnancy, the perinatal period and older age are critical periods, when more antibiotics and more anti-ulcer drugs are prescribed. Therefore, (1) their usage should be carefully considered and limited to precise indications and (2) the development of methods reconstituting the microbiota after drug-induced dysbiosis, such as probiotics and micronutrients, is urgently required.

### Asthma prevention

#### I) Probiotics

According to the World Health Organization (WHO), 2001, probiotics are defined as live microorganisms that, when administered in adequate amounts, confer a health benefit to the host [137]. Their efficacy in asthma prevention and treatment is not absolutely clear at the moment, however, a recent meta-analysis of RCTs in six databases showed in long-term follow-up that the administration of *Lactobacillus rhamnosus* (LGG) facilitated the prevention of asthma [138].

Bacteria used for probiotics mainly belong to lactic acid bacteria (Phylum *Firmicutes*, order *Lactobacillales*, *genus Lactobacillus*, *Streptococcus*, *Enterococcus*), to *Actinobacteria* (order *Bifidobacteriales*, genus *Bifidobacterium*), and non-pathogenic *Escherichia coli* (reviewed in [139]). These bacteria exert a number of positive effects, e.g., a direct effect on the maturation of the gut barrier (intestinal mucosa structure/function) and on the development of tolerogenic dendritic cells, which later on influence the local intestinal as well as the systemic immune response (reviewed in [140]). Thus, counteracting microbiome dysbiosis in allergy and asthma using probiotics seems worthwhile [141].

However, also bacterial products may be beneficial. For example, short-chain fatty acids produced in the digestive tract suppress allergic airway inflammation by modulating DC progenitors. Especially, butyrate has potent effects and together with propionate can induce tolerogenic DC which then enhance Tregs [142].

Among several promising studies in asthma animal models, Wu et al. tested the effect of LGG in an ovalbumin (OVA) asthma-mouse model [143]. The animals treated orally with LGG for 2 weeks, either as pre- or post-sensitization treatment, showed lower airway resistance, inflammatory cell numbers, and Th2-cytokines in the lung while Th1- and Treg-cytokines were elevated. The authors concluded that oral probiotics might be an additional or supplementary therapy to other clinical allergy/asthma therapies. In other mouse models, *L. reuteri* supplementation increased Tregs in splenocytes [144] and attenuated major characteristics of an asthmatic response, including airway eosinophilia, local cytokine responses, and hyperresponsiveness to methacholine [145]. Interestingly, only living bacteria were able to evoke these results.

Regarding the optimal time point of probiotics application, perinatal intragastric application of LGG in a BALB/c mouse model resulted in intragastric colonization of mothers. The offspring showed reduced expression of TNF-α, IFN-γ, IL-5, and IL-1 in splenocytes, and reduced allergic airway and peribronchial inflammation in the lung [146]. In a recent OVA-induced asthma mouse model, probiotic oral treatment with combined *Lactobacilli casei/lactis/acidophilus* plus *Bifidobacteria bifidium*/*lactis* reduced allergic airway disease when given perinatally [147]. In gut microbiota, higher *Firmicutes* and *Actinobacteria* appeared when probiotics were applied at neonatal age, accompanied by higher CD4+ Treg-cell numbers in BALF and increased caecal butyrate, whereas only *Actinobacteria* were significantly higher compared to sham-treated mice when probiotics were applied to adult animals. This paper also proved in transfer experiments that the window of opportunity as well as the efficacy of T-cells are most pronounced at neonatal age. Accordingly, probiotics *Bifidobacterium lactis* or *Lactobacillus rhamnosus* LGG orally applied to neonate mice suppressed all aspects of the asthmatic phenotype [148].

Coming enthusiastically from these animal studies into the human system, the application of probiotics to high-risk children in the first 6 months of life did not show any significant differences for asthma incidence at the age of 5 years, although a trend was found with 17.4% affected by asthma in the control group vs. 9.7% in the LGG-treated children [149].

When 4 to 10-year-old children with atopic asthma were given oral probiotics (*Lactobacillus acidophilus*, *Bifidobacterium bifidum*, *Lactobacillus delbrueckii* subsp. *Bulgaricus*) (Trilac) for 12 weeks [150], the children receiving the probiotic had significantly improved lung function, less episodes of asthma exacerbations, and reduced amount of use of bronchodilatators than the children on placebo. In PBMCs, statistically significant increase in the expression of HLA-DR on monocytes and decrease of CD8CD45RA+ lymphocytes were observed in the Trilac group.

Finally, Huang et al. showed that *Lactobacillus paracasei* (LP), *Lactobacillus fermentum* (LF), or their combination, given to asthmatic children aged 6 to18 years as capsules for 3 months, lowered the asthmatic severity, improved asthma control, increased the peak expiratory flow rate, and decreased IgE levels [151]. The combination of both strains appeared to be most effective.

An 8-week randomized trial in children with asthma and allergic rhinitis treated with *Lactobacilllus gasseri* showed a significant reduction in symptoms along with improvement of pulmonary function [152].

A meta-analysis of human studies from 2013 summarizes that probiotics prenatally or given in early-life reduce the risk of atopic sensitization and decrease total IgE levels in children, however, from different study set-ups, it was concluded that they may not reduce the risk of asthma/wheeze [153]. Also, database reviews concluded that the current evidence is much stronger for probiotics being effective in eczema and allergic rhinitis rather than in prevention [154] or treatment of asthma [132, 155, 156]. Contrasting this, a recent meta-analysis evaluating also sub-groups for application of specific strains suggested that supplementation with explicitly LGG in the postnatal period may be beneficial in asthma prevention [138]. At the moment, it is stated by international health organizations and allergy organization guidelines that more studies are needed on whether asthma/wheezing prevention with probiotics is effective, as studies to date may “not have used the right probiotic, the right dose, the right timing or duration and/or population” [157, 158]. Also, geographic, cultural, and nutritional habits might differ widely. Therefore, more research is needed to develop clear-cut recommendations for asthma prevention and treatment with probiotics, and they need to carefully focus on specific probiotic strains or cocktails thereof and on assessment of the outcome in a longer follow-up [158].

#### II) Bacterial lysates

Respiratory tract infections (RTIs) occur very often in young children and may be a prerequisite for development of recurrent wheeze and asthma. Next to vaccination strategies to prevent RTIs, the use of nonspecific immunomodulators derived from bacterial respiratory tract pathogens has gained attention [159]. In contrast to probiotics, bacterial lysates have transient effects only and are not living.

The composition of such bacterial lysates include species that have been described to be present in dysbiotic microbiota of children that were prone to develop recurrent wheeze and asthma later in life [31], among them *Haemophilus influence*, *Moraxella catarrhalis*, and *Streptococcus pneumoniae*. In a recently published retrospective study, 200 children up to 6 years with a history of recurrent respiratory tract infections received OM-85 (Broncho-Vaxom®), which consists of alkaline lysis of 21 bacterial strains of eight species of respiratory tract pathogens, via the oral route over 2 years [160]. The treatment resulted in significantly lower RTIs, wheezing episodes and intake of antibiotics, and reduction of new infective episodes in children with recurrent RTIs. One of the key mechanisms of this compound may be attributed to the connection between gut and lung, the aforementioned gut-lung axis. Navarro and colleagues described in a mouse model of allergic airway disease that orally administered OM-85 activated gut DCs which induced trafficking of Treg cells to the lung [161]. The clinical efficacy was presented in several studies showing that administration of OM-85 was able to alleviate RTIs and to reduce asthma exacerbations in children with recurrent wheeze [162, 163]. Similarly, the administration of a polyvalent mechanical bacterial lysate tablet (PMBL®) via the sublingual route to 6 to 16-year-old children with allergic asthma led to a significant reduction of asthma exacerbations [164]. Thus, bacterial compounds seem to reduce RTIs and may influence asthma via immunoregulatory mechanisms that are induced in the gut but can become evident in reduced lung inflammation and hyper-reactivity. However, the influence of such bacterial lysates on resident airway microbiota, especially early in childhood, still needs to be investigated in detail [165].

## Conclusion

There is growing evidence that the microbiome of the airways plays a major role in the development of asthma. In early childhood, the establishment of a highly diverse, non-pathogenic bacterial community seems important, but simultaneously this is a so-called window of opportunity to manipulate the upper airway microbiota and the immune system that could prevent the development of asthma in children. Airway microbiome dysbiosis contributes to asthma pathogenesis and severity in adults. Microbial composition in lung and gut can be influenced by several environmental factors. Environmental stress factors such as allergens, pollution, viral infection and use of antibiotics or PPIs can cause bacterial dysbiosis and promote asthma. On the other hand, exposure to varied bacterial components, but also proteins in a farm environment, could be protective against the development of asthma. Preventive and therapeutic management to counteract microbiome dysbiosis and restore a healthy microbiome by probiotics, fecal microbiota transplants or bacterial lysates has not arrived in clinical routine so far. Thus, further mechanistic studies are needed to explore the influence of microbial composition on asthma pathogenesis, especially in the lung, to subsequently refine treatment regimens that can prevent airway diseases.
